# Nursing Education for Sustainable Development: A Concept Analysis

**DOI:** 10.1002/nop2.70058

**Published:** 2024-10-18

**Authors:** Dorothy Serwaa Boakye, Atswei Adzo Kwashie, Samuel Adjorlolo, Kwadwo Ameyaw Korsah

**Affiliations:** ^1^ Department of Health Administration and Education University of Education Winneba Ghana; ^2^ School of Nursing and Midwifery, College of Health Sciences University of Ghana Accra Ghana

**Keywords:** concept analysis, curriculum integration, nursing education, sustainable development

## Abstract

**Background:**

Sustainability represents an increasingly vital yet inconsistently implemented topic in nursing education. Formal concept analysis can promote unified conceptualisation to inform sustainability integration in nursing curriculums.

**Objective:**

Analyse the concept of ‘nursing education for sustainable development’ using the Walker and Avant framework to clarify meaning and application in nursing programmes.

**Method:**

The literature was systematically searched for attributes, antecedents and consequences used to formulate a concept definition, and compared to existing conceptualisations.

**Results:**

Core concept attributes are interconnectedness of human/ecological health, sustainability‐focused curriculums, competency cultivation and social justice orientations. Antecedents include recognising ecological determinants of health and committed nursing faculty. Consequences encompass the preparation of sustainability‐competent nurses and elevated nursing contributions to sustainable health systems.

**Conclusion:**

The concept analysis provides an original synthesised perspective advancing coherence and applicability to guide sustainability education in nursing programmes through a translational framework for competency, curriculum design and content delivery approaches.

**Reporting Method:**

We followed the guidelines outlined in the Walker and Avant framework in the conduct and reporting of this paper.

**Patient or Public Contribution:**

No patient and public contribution.

## 1 Introduction

Sustainability has become an increasingly crucial issue across industries, including healthcare and nursing education (Goodman 2011; Sendall, Fleming, and Lidstone 2011). Educating future generations of nurses on sustainability supports environmentally responsible policies and practices in healthcare for the long term (Joseph et al. 2021). However, there remain gaps regarding the consistent conceptualisation and integration of sustainability in nursing curriculums (Lawlis et al. 2014). To advance a shared understanding in this emerging field, formal concept analysis methodology can be highly useful for constructing conceptual clarity (Walker and Avant 2005). The Walker and Avant approach to concept analysis is one of the most established methods that involves identifying essential attributes, antecedents, consequences and prototypes to develop a sdefinitive conceptualisation of the concept (Rodgers, Jacelon, and Knafl 2018).

The purpose of this concept analysis using the Walker and Avant method is to produce a nursing‐focused conceptual definition and framework of sustainability education for undergraduate nursing programmes. The research question is: What are the core attributes, prerequisites, results and exemplars that represent the concept of ‘nursing education for sustainable development’? By rigorously analysing this concept, the resulting theoretical understanding can help inform nursing curriculum development, advance consistency in nursing sustainability competencies, and ultimately guide the integration of sustainability content in nursing schools towards sustainable healthcare systems.

## 2 Background

Integrating sustainability principles into nursing education aligns with longstanding international policies emphasising nursing's role in primary health care and addressing social determinants of health. The 1978 Alma‐Ata Declaration first highlighted nursing's crucial responsibilities in mitigating economic and social conditions impacting community well‐being. It laid the groundwork for an expanded population health focus beyond just clinical treatment (WHO 1978). The 1986 Ottawa Charter for Health Promotion (WHO 2024) similarly urged preparing health professionals like nurses to confront the interconnected economic, social and environmental factors allowing poor health to persist—foreshadowing today's need for sustainability competencies.

Over subsequent decades, landmark policies continued positioning nurses to holistically promote equitable communities resilient to modern challenges. The 2015 sustainable development goals (SDGs) codified an agenda to collectively advance people, the planet, prosperity, peace and partnership through cross‐sector collaboration (Upvall and Luzincourt 2019). Achieving the SDGs necessitates developing a health workforce, including sustainability‐oriented nurses, capable of cultivating sustainable, just communities able to withstand threats like climate change, conflicts and globalisation (McKinnon and Fitzpatrick 2017; Rosa et al. 2019; Rosa and Coach 2017).

Nursing has been identified as a pivotal profession to the United Nations agenda towards the achievement of the SDGs with a demonstrable alignment between health and the seventeen (17) goals (International Council of Nurses 2017; World Health Organization 2016). Therefore, nursing education plays a crucial role in promoting sustainable development (Benton and Ferguson 2016). Integrating sustainability content across both the theoretical foundations and applied practice experiences in nursing education programmes is vital. Educators can cultivate sustainable change agents capable of meaningfully contributing nursing's unique perspectives and skillsets towards SDG targets across ecological, economic and social domains. This bidirectional integration prepares future nurses as sustainability leaders improving health and well‐being for current and future generations (Lilienfeld et al. 2018; WHO 2020; Fields et al. 2021).

Sustainability has garnered increasing attention in nursing curriculums and education in recent years (Thompson and Schwartz Barcott 2017). A study by Sendall, Fleming, and Lidstone (2011) traces the increasing focus on sustainability within nursing, highlighting conceptualisations that recognise the connections between environmental health, human health and nursing advocacy. However, analyses suggest there remains a lack of consensus around sustainability competencies and policy in nursing education (Lawlis et al. 2014; Mitchell 2011).

Existing literature reveals variable conceptualisations of sustainability in nursing education contexts. Thompson and Schwartz Barcott (2017) focus strongly on environmental health, defining sustainability for nurses primarily through an ‘environmental lens’ with emphasis on climate change impacts. Meanwhile, Brundiers et al. (2021) conceptualise four pillars of sustainability in nursing: economics, environment, society/culture and governance, arguing for a blended integration across these areas.

Recent qualitative studies provide additional perspectives into how nursing educators and nursing students conceptualise these issues about curricular priorities (Anåker et al. 2021; Kitt‐Lewis et al. 2020; Richardson et al. 2014). Richardson et al. (2016) found notions of social justice and advocacy were salient themes among nursing faculty, whereas environmental stewardship resonated more among nursing students. Still, other conceptualisations align sustainability in nursing curricula closely with wider organisational and policy dimensions in healthcare settings (Roden and Lewis 2021; Fields et al. 2021).

Synthesising these works highlights gaps and inconsistencies in conceptualising nursing education for sustainability. As Richardson et al. (2016) argue, ambiguity persists around constructs like environmental health literacy versus wider interpretations of sustainability competencies expected of nurses. This demonstrates the need to advance conceptual clarity through structured concept analysis geared for application in nursing sustainability education contexts specifically (Rodgers, Jacelon, and Knafl 2018; Walker and Avant 2005).

## 3 Methods

The Walker and Avant (2011) model of concept analysis is a widely recognised framework used in nursing and other healthcare disciplines to explore and clarify the meanings of complex concepts. Developed by Kay C. Avant and Lorraine O. Walker, this model provides a systematic and structured approach to concept analysis, allowing researchers and practitioners to explore complex concepts in a rigorous and comprehensive manner. Per Walker and Avant (2011), this inductive process derives concept meaning through an iterative cycle of scholarly literature review and analytical scrutiny of cases and related examples. Using this well‐established method provides a systematic foundation for conceptual clarity and real‐world applicability. It also helps in promoting clarity and consistency in understanding concepts, enhancing communication and supporting evidence‐based practice in healthcare and other disciplines. The model has eight steps used to highlight the aspects and attributes of a concept. These eight steps are elaborated in Table 1.

**TABLE 1:** Walker and Avant model of concept analysis: analysis steps.

Steps	Subtarget	Description
1	Select a concept	Selecting a concept for analysis is ideally based on the author's personal interest and expertise or chosen because it is deemed essential for the research at hand
2	Determine aims/purposes of the concept analysis	Often, the aim is to explore the current meanings, uses, characteristics and relationships of a concept in the contexts of research and practice in a specified discipline in order to develop an operational definition
3	Identify uses of the concept	This involves an indepth integrative literature review to identify how the concept is delineated, applied or related across various disciplines and sources
4	Determine the defining attributes	The data from step 3 are analysed to determine the core, clustered attributes or characteristics most commonly associated with the concept
5	Construct a model case	A model case is constructed that contains all the defining attributes and illustrates a real‐life exemplary instance of the concept
6	Construct additional cases	Additional cases are then constructed to illustrate related instances, borderline examples or contrary cases lacking core attributes
7	Identify antecedents and consequences	Antecedents are identified as the events or circumstances preceding the concept's occurrence. Consequences are the events resulting from the concept's presence
8	Define empirical referents	The final step identifies the empirical referents or real‐world observable phenomena that demonstrate the existence of the concept. It also describes how the concept is utilised in research and in practice

**FIGURE 1:**
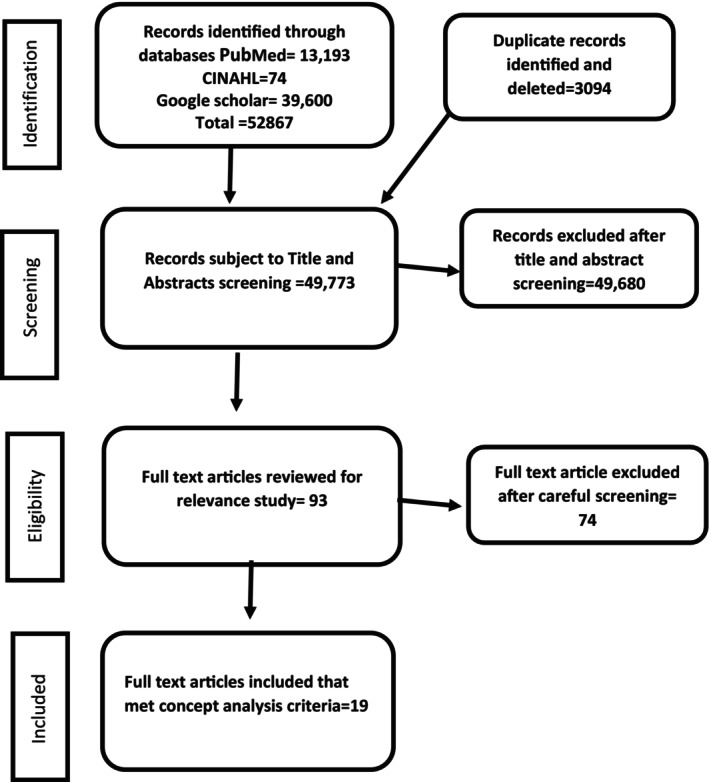
PRISMA flow diagram.

Literature was systematically searched in PubMed and CINAHL databases in November 2023 using the key terms “nurs* education,” “sustainab* development goal*,” “sustainability principle*” “sustainable development” “curricul*,” “competen*”. Additional highly relevant sources were snowball sampled from references and relevant journal websites. Rigorous Preferred Reporting Items for Systematic Reviews and Meta‐Analyses (PRISMA) methods were applied during screening and review (Moher et al. 2009; Page et al. 2021). The studies for this concept analysis were selected based on predetermined criteria for inclusion and exclusion. The inclusion criteria were primary research studies (all designs) that explore, describe or measure concepts/attributes related to sustainability education in nursing programmes, systematic reviews, meta‐analyses and other reviews synthesising evidence on sustainability integration in nursing curricula, theoretical/conceptual papers and frameworks focused on defining or modelling sustainability competencies for nurses, sources from all geographical regions and publications in English language. Studies/papers focused solely on environmental health nursing without broader sustainability framing, sources about generic ‘environmental education’ without explicit nursing context and opinion pieces, commentaries or editorials without theoretical/empirical analysis were excluded. Our database searches and Google Scholar exploration yielded 52,867 records. Following an assessment of their eligibility, we pinpointed 93 studies for full‐text screening, of which 19 aligned with the inclusion criteria. Details of the screening process is outlined in Figure 1.

The majority of the articles included in this concept analysis were primarily of secondary sources, such as concept analysis (1), scoping reviews (2), narrative review (6), discussion papers (4) and integrative review (1). Primary sources were scarce, with only four articles conducting original research (5). The included studies underwent thorough quality appraisal by two independent reviewers using standardised critical appraisal tools. The specific tool utilised depended on the study design. Narrative reviews and discussion papers were appraised with the scale for the assessment of narrative reviews (SANRA) (Baethge et al. 2019), scoping reviews and integrative reviews were appraised with the Joana Briggs Institute critical appraisal checklist for systematic reviews and research synthesis (JBI 2014), Survey studies were appraised with JBI critical appraisal tool for cross‐sectional studies (Moola et al. 2017) and mixed‐method studies were appraised with the mixed‐method appraisal tool (Hong et al. 2018).

For concept attribute extraction, a directed qualitative content analysis methodology as described by Hsieh and Shannon (2005) was utilised. A coding schema was developed by two independent coders based on Walker and Avant's (2005) concept analysis framework. A summary of data extracted for this concept analysis is provided in Table 2.

**TABLE 2:** Summary of data extracted.

References	Publication title	Study design	Sample characteristics	Uses of the concept	Attributes	Antecedents	Consequences	Empirical referents	Methodological rating
Clark et al. (2020)	Sustainable Healthcare Elective in Nursing: A futures‐thinking approach	Discussion paper	Not applicable	The concept discussed is sustainable healthcare within nursing education It emphasises embedding sustainability principles into nursing curricula	Ecology, Holism, Interdisciplinary collaboration				5/12
Smith et al. (2020)	Occupational Therapy and Environmental Sustainability: A Scoping Review	Scoping review	Not applicable	Occupational therapists are expected to intervene at individual, group, and population levels to address concerns arising from climate change, such as food availability, displacement, and physical and mental health effects of extreme weather events	Increased awareness and advocacy for sustainability and health within the profession				7/11
Anåker and Elf (2014)	Sustainability in nursing: a concept analysis	Concept analysis	Not applicable		Holism, Globalism, Future	Willingness to Change	Participation in Sustainable Development	Ecological, global, environmental, and holistic knowledge among nurses can be measured through observations or interviews. Surveys and focus‐group interviews can assess motivation to work sustainably. Environmental impact can be measured by observing resource usage and CO_2_ emissions	
Roden and Lewis (2021)	Looking beyond Nursing Education Practice to Include Sustainable Health‐Care Systems Processes	Discussion paper	Not applicable	The concept involves incorporating sustainable practices into hospital operations to reduce greenhouse gas emissions and mitigate climate change effects	Increased awareness and advocacy for environmental sustainability among healthcare professionals	Awareness of the need for sustainable healthcare systems to protect environmental and human health			6/12
Luque‐Alcaraz et al. (2024)	The environmental awareness of nurses as environmentally sustainable health care leaders: a mixed method analysis	Mixed method	314 registered nurses for the quantitative survey and 14 for the qualitative study	The concept of nursing environmental behaviour and knowledge is utilised to assess nurses' awareness, knowledge, attitudes, and skills regarding environmental sustainability in healthcare settings	Environmental awareness	Provision of formal training and courses on environmental sustainability for nursing staff	Enhanced environmental awareness and knowledge among nursing staff		12/17
Goodman (2011)	The need for a ‘sustainability curriculum’ in nurse education	Review paper	Not applicable		Interconnectedness between human health, ecological health, and socio‐political environments	Unsustainable economics, lifestyles, and consumption patterns contribute to climate change and ecological degradation	Addressing carbon emissions, climate change, and unsustainable living is essential for safeguarding health		7/12
Goodman and East (2013)	The ‘sustainability lens’: A framework for nurse education that is ‘fit for the future’	Review paper	Not applicable	It discusses the need for addressing sustainability and climate change in nurse education	Curriculum development		Developing a sustainability lens in nursing education		8/12
Rosa et al. (2019)	Nursing and midwifery advocacy to lead the United Nations Sustainable Development Agenda	Review paper	Not applicable	The concept is used to illustrate the interconnectedness of the Sustainable Development Goals (SDGs) with health outcomes	Interdisciplinary collaboration	Addressing complex global challenges	Promoting social justice and sustainability, Awareness of global trends		8/12
Sensor et al. (2021)	Nurses Achieving the Sustainable Development Goals: The United Nations and Sigma	Discussion paper	Not applicable			Partnership and collaboration	Leadership, shared governance and advocacy		8/12
Goodman (2016)	Developing the concept of sustainability in nursing	Review paper	Not applicable		Globalism, Holism	Socio‐political context in nursing education	A deeper understanding of sustainability		7/12
Bryne et al. (2013)	Exploring sustainability themes in engineering accreditation and curricula	Mixed method study	Engineering academics and professional who took part in workshop conference on sustainability		Incorporation of sustainable practices into nursing curriculum		Improved environmental sustainability in healthcare settings, Enhanced social responsibility among nursing professionals		9/17
Argento et al. (2020)	Integrating sustainability in higher education: a Swedish case	Narrative Case focused on Kristianstad University in Sweden	Not applicable		Holistic care and ecological perspectives	Faculty interest and participation, Interconnectedness between nursing, health, and sustainability	Enhanced understanding of holistic care and its relation to sustainability, Improved health outcomes through sustainable nursing practices		8/12
Ubochi et al. (2019)	Building a strong and sustainable health care system in Nigeria: The role of the nurse	Discussion paper	Not applicable		Leadership and advocacy skills, Commitment to inter‐professional collaboration	Ethical responsibilities outlined in professional codes of conduct, Advocacy and collaboration efforts	Increased nursing involvement in policy‐making and planning, Enhanced primary healthcare delivery and community engagement		7/12
Howell (2021)	Engaging students in education for sustainable development: The benefits of active learning, reflective practices and flipped classroom pedagogies	Survey study	Students enrolled on the course in 2016 and 2017			Student engagement and participation	Enhanced student engagement and participation in ESD		5/8
Bvumbwe and Mtshali (2018)	Nursing education challenges and solutions in Sub Saharan Africa: an integrative review	Integrative review	Not applicable		Collaboration and partnerships, Curriculum reforms, Strategic leadership, networking and partnership	Infrastructure and resources			7/11
Sherman et al. (2020)	The Green Print: Advancement of Environmental Sustainability in Healthcare	Narrative review	Not applicable		Systems re‐thinking and performance feedback, Establishing research priorities to improve the environmental performance of healthcare services	Globalisation Ecology Holistic healthcare	Environmental Sustainability in Clinical Care Advocacy for policy and practice	Integrating environmental performance metrics into existing reporting structures	8/12
Fields et al. (2021)	Nursing and the Sustainable Development Goals: A Scoping Review	Scoping review	Not applicable		Globalisation and Global Health Workforce	Education and awareness about the SDGs among nurses	Representation of nurses in policy and decision‐making processes Nurses' ability to contribute to the SDGs		7/11
Huss et al. (2020)	Education for sustainable health care: From learning to professional practice	Review paper	Not applicable		Early introduction and integration of content in the curriculum Multi‐disciplinary collaboration Skills application to clinical practice	Education and awareness about the SDGs and environmental sustainability Adaptable educational resources	Challenging unsustainable practices in the work environment		8/12
Richardson et al. (2016)	Including sustainability issues in nurse education: A comparative study of first year student nurses' attitudes in four European countries	Comparative survey design.	916 nursing students (UK: 450, Germany: 196, Spain: 124, Switzerland: 146)		Integration of sustainability in nursing curricula	Availability of resource materials for sustainable education Sustainability and climate change as a global challenge		Competency and clinical skills on sustainability was assessed with objective structured clinical examination (OSCE). Sustainability attitudes among nursing students could be assessed by the 7 item sustainability attitude in nursing survey (SANS) questionnaire	6/8

Exemplar cases were drawn from retained studies assessed as sufficiently describing or demonstrating the concept attributes. These cases provide a real‐world illustration of the conceptual meaning (Walker and Avant 2005). As recommended by Walker and Avant (2005), contextually diverse cases were intentionally described as explicitly addressing sustainability in nursing education.

## 4 Results

### 4.1 The Concept

In this instance, the concept was ‘nursing education for sustainable development’.

### 4.2 Aim/Purpose

This concept analysis aimed to explore, describe, explain and clarify the concept, nursing education for sustainable development as well as explore its attributes, and understand its relationships. We also sought to provide a definition that facilitates the use and understanding of the concept in nursing.

### 4.3 Identify Uses of the Concept

To identify the uses of the concept ‘nursing education for sustainable development’, we explored how this concept is employed in nursing literature, theory, practice and education. Here are some potential uses of the concept:

*Curriculum Development*: According to Clark et al. (2020), incorporating sustainability concepts in nursing curricula prepares future nurses to address the environmental, social and economic determinants of health, contributing to sustainable development.

*Competency Development*: The International Council of Nurses (ICN) identifies nursing education for sustainable development as essential for developing competencies related to environmental health, resource management and advocating for sustainable policies (International Council of Nurses 2017). This involves equipping nurses with the knowledge, skills and attitudes necessary to promote environmental sustainability, address social determinants of health and advocate for sustainable healthcare systems.

*Research and Evidence‐Based Practice*: The concept of nurse education for sustainable development can guide research endeavours focused on understanding the impact of sustainable practices on nursing outcomes, patient health and healthcare systems. For instance, Smith et al. (2020) conducted a scoping review on the implementation of sustainable practices in occupational therapy and found that education for sustainable development was crucial in promoting sustainable behaviours and improving patient outcomes.

*Professional Ethics and Responsibility*: The American Nurses Association (ANA) highlights nurse education for sustainable development as a means to cultivate nursing professionals' ethical responsibilities towards environmental sustainability and to enhance the quality of healthcare (American Association of Colleges of Nursing 2020). This includes promoting environmentally friendly practices, reducing waste and advocating for sustainable policies and practices within healthcare settings.

*Policy and Advocacy*: The concept can be employed to inform policy development and advocacy efforts aimed at integrating sustainability principles into healthcare systems. Nursing education for sustainable development equips nurses to be influential advocates for sustainable healthcare policies and practices at local, national and global levels. (Rosa et al. 2019).

### 4.4 Conceptual Definition

Integrating these components, nursing education for sustainable development is defined as follows: The process of preparing nursing students to understand the interconnectedness of human and environmental health and to address sustainability issues in healthcare, with a focus on enacting systems thinking, social justice orientation and environmentally responsible practices throughout their nursing careers (Anåker and Elf 2014).

This moves beyond previous single‐dimension conceptualisations focused predominantly on environmental learning, towards a more comprehensive characterisation aligning with transformational sustainability pedagogies in higher education (Cotton and Winter 2010). It purposefully integrates dimensions of sustainability competency development as well as antecedent factors and project outcomes reflecting wider constructs of sustainability in nursing education contexts (Roden and Lewis 2021; Evans, 2019). According to the International Council of Nurses (2017), this concept acknowledges that nurses play a vital role in fostering sustainable healthcare systems and advocating for policies and practices that promote environmental sustainability, social equity and improved health outcomes.

### 4.5 Identify the Attributes

According to Walker and Avant (2011), attributes are the features or qualities that must be present for the concept to be considered nursing education for sustainable development. The essential characteristics or attributes of this concept discovered in the literature are as follows:

- Environmental Awareness: Nurse education for sustainable development emphasises the importance of understanding environmental issues and their impact on health. Nurses should be aware of environmental factors that contribute to health problems and be equipped to promote sustainable practices to mitigate these issues (Luque‐Alcaraz et al. 2024).
- Interdisciplinary Approach: This concept recognises the need for collaboration and integration of knowledge from various disciplines. Nurse education for sustainable development encourages interdisciplinary learning, involving fields such as public health, environmental science, ethics and social sciences, to provide a comprehensive understanding of sustainable healthcare (Clark et al. 2020; Huss et al. 2020).
- Holistic Perspective: Nurses need to adopt a holistic perspective that considers the interconnectedness of ecological, social and economic systems. They should understand how sustainable practices can contribute to improved health outcomes for individuals, communities and the planet as a whole (UNESCO 2017; Sherman et al. 2020).
- Ethical Considerations: Nurse education for sustainable development emphasises ethical decision‐making and the promotion of ethical practices in healthcare. This includes addressing issues such as environmental justice, resource allocation, equitable access to healthcare and responsible use of resources (Goodman and East 2013).
- Systems Thinking: Nurses should develop the ability to think critically and systemically, understanding the complex relationships between healthcare, the environment and society (Center for sustainable Future 2008).
- Advocacy and Leadership: Nurses should be equipped with the skills to advocate for sustainable healthcare practices and lead initiatives for change. This includes raising awareness, influencing policies and collaborating with stakeholders to promote sustainable development in healthcare settings (Sensor et al. 2021; Smith et al. 2020).
- Lifelong Learning: Nursing education for sustainable development acknowledges the evolving nature of sustainability challenges. It emphasises the need for nurses to engage in lifelong learning, staying updated on current research, best practices and emerging issues related to sustainability in healthcare (Goodman 2011).
- Practical Application: This concept emphasises the practical application of sustainable practices in nursing education. It encourages hands‐on learning experiences, such as clinical placements in sustainable healthcare settings or participation in sustainability projects, to enhance nurses' skills and understanding of sustainable development (Goodman and East 2013; Huss et al. 2020).

### 4.6 A Model Case

This hypothetical case of a nursing education programme for sustainable development is created by the authors. A group of nursing students from the University of Africa (pseudonym) is enrolled in a community health course dubbed ‘Green Impact for Health’ programme implemented by the nursing school in the University of Africa in collaboration with a local hospital, Serwaa's Memorial Hospital.

In this programme, the nursing students are engaged in a series of workshops and training sessions that promote sustainable healthcare practices. They learn about waste management, energy conservation, water conservation and other eco‐friendly strategies relevant to healthcare settings.

The students are then divided into teams and assigned to the different units in the hospital where they apply their knowledge to identify areas for improvement. They conduct energy audits, assess waste management systems and suggest sustainable alternatives. For example, they may recommend the use of energy‐efficient equipment, recycling programmes, or the implementation of electronic medical records to reduce paper waste.

Throughout the programme, the students collaborate with hospital staff, including nurses, administrators and environmental services, to implement their recommendations. They monitor the impact of their interventions and make adjustments based on feedback and data analysis.

The ‘Green Impact for Health’ programme not only equips nursing students with knowledge and skills related to sustainable healthcare but also empowers them to become agents of change within the healthcare system. It highlights the importance of practical application, interdisciplinary collaboration and leadership in promoting sustainable development in nursing practice.

### 4.7 Borderline Case

This borderline case is a fictitious scenario created by the authors.

A nursing school at the University of Africa (Pseudonym) recognises the importance of sustainability in healthcare and establishes a collaboration with a nearby hospital that has embraced sustainable practices. They develop a joint programme where nursing students are assigned to clinical rotations in specific units of the hospital that prioritise sustainability.

During their clinical placements, nursing students observe and actively participate in sustainable healthcare practices. They learn about the hospital's waste management system, energy‐efficient equipment and conservation efforts. They also witness sustainable initiatives like recycling programmes, water conservation strategies and the use of renewable energy sources.

The nursing school integrates sustainability‐focused modules into the core curriculum, ensuring that all nursing students receive dedicated education on sustainable development. These modules cover topics such as environmental health, climate change and health, sustainable resource management and ethical considerations related to sustainability in healthcare.

To further enhance the learning experience, the nursing school invites guest speakers from environmental organisations, public health agencies and sustainability experts to share their knowledge and experiences with the students.

Throughout their education, nursing students engage in research projects and community outreach activities related to sustainable healthcare. They collaborate with faculty members, hospital staff and local communities to raise awareness about sustainable practices, advocate for policy changes and develop innovative solutions to address sustainability challenges.

Although the nursing school has taken significant steps to integrate sustainability into its curriculum and provide practical application opportunities, there is an ongoing effort to expand the scope of sustainability education, foster interdisciplinary collaborations and continuously improve sustainable practices within the nursing school and clinical settings as well as provide continuous training for faculty members.

### 4.8 Contrary Case

In a contrary case of nursing education for sustainable development, a nursing school in the University of Africa (Pseudonym) completely neglects the integration of sustainability principles into their curriculum and fails to recognise the importance of sustainable development in nursing practice.

This nursing school follows a traditional curriculum that solely focuses on clinical skills, medical knowledge and patient care without addressing sustainability as a core component. The school does not offer any dedicated courses or modules related to sustainable healthcare practices, environmental health or resource management. The faculty members are not knowledgeable about sustainable development and do not actively promote or advocate for sustainability in healthcare. There is a lack of awareness among the faculty regarding the potential impact of healthcare practices on the environment, society and future generations.

This nursing school does not collaborate with external organisations or healthcare facilities that prioritise sustainability. There are no opportunities for students to gain practical experience or exposure to sustainable healthcare practices during their clinical placements.

In this contrary scenario, nursing education completely disregards the integration of sustainability principles, hindering the preparation of nurses to address the environmental, social and economic challenges in healthcare.

### 4.9 Identify Antecedents and Consequences

The occurrence or development of the concept ‘nursing education for sustainable development’ is influenced by several factors. These factors can vary in their impact and significance across different contexts and educational settings. Some of the key factors include: *Institutional Priorities*: Institutions that prioritise sustainability and recognise its importance in healthcare are more likely to integrate sustainable development principles into their nursing curricula (Goodman 2016). *Educational Policies and Accreditation Standards*: When sustainability is explicitly included as a requirement or expectation in accreditation standards or educational guidelines, nursing programmes are more likely to incorporate sustainable development concepts into their curricula (Byrne et al. 2013). *Faculty Expertise and Interest*: The knowledge, expertise and interest of faculty members play a significant role in shaping the occurrence and development of nurse education for sustainable development (Argento et al. 2020). *External Partnerships and Collaborations*: Collaborations between nursing schools and external organisations, such as healthcare facilities, environmental agencies or sustainability‐focused institutions, can greatly influence the occurrence and development of nurse education for sustainable development (Ubochi et al. 2019). *Student Interest and Engagement*: When students demonstrate a strong interest in sustainability and advocate for its inclusion in the curriculum, nursing schools are more likely to respond and adapt their educational programmes accordingly (Howell 2021). *Societal and Global Context*: The societal and global context, including the recognition of environmental challenges, climate change, and the United Nations SDGs, can influence the occurrence and development of nursing education for sustainable development (The Center of Sustainable Future 2008). *Resource Availability*: The availability of resources, including funding, infrastructure, teaching materials and faculty development opportunities, can impact the occurrence and development of nursing education for sustainable development (Bvumbwe and Mtshali 2018).

The concept of ‘nursing education for sustainable development’ can lead to various consequences that have a positive impact on healthcare, the environment and society. Some of the key outcomes that emerge as a result of this concept include: increased awareness and understanding (Luque‐Alcaraz et al. 2024), integration of sustainable practices into healthcare (Goodman 2016), improved health outcomes (Sherman et al. 2020), ethical decision‐making in healthcare (Goodman 2011) fosters the development of advocacy and leadership skills among nurses (Sensor et al. 2021), research and innovation and contribution to SDGs (Goodman 2011). A summary of the attributes, antecedents and consequences of this concept analysis is outlined in Table 3.

**TABLE 3:** Summary: attributes, antecedents and consequences.

Attributes	Antecedents	Consequences
Environmental awareness	Institutional priorities	Increased awareness and understanding
Interdisciplinary approach	Educational policies and accreditation standards	Integration of sustainable practices into healthcare
Holistic perspective	Faculty expertise and interest	Ethical decision‐making in healthcare
Ethical considerations	External partnership and collaborations	Development of advocacy and leadership skills among nurses
Systems thinking	Student interest and engagement	Research and innovation and contribution to Sustainable development goals
Advocacy and leadership	Societal and global context	
Practical applications	Resource availability	
Lifelong learning		

### 4.10 Empirical Referents

Empirical referents for sustainable development in research or practice, provide several measurable indicators or variables to be considered. These indicators can help evaluate the extent to which nursing education programmes incorporate sustainable development principles and assess the outcomes or impact of such education (Anåker and Elf 2014).

The attributes, antecedents and consequences of sustainability development could be assessed through various approaches. For instance, faculty and student learning competencies could be assessed through self‐efficacy scales, self‐reported measures, interviews and pre and posttest. Curriculum integration could be evaluated by assessing the number of dedicated courses and percentage of total curriculum courses. Practical application of sustainability principles and clinical skills could be measured with observation or evaluation of students' application of sustainable healthcare practices during clinical rotations or simulations or an assessment of students' proficiency in implementing eco‐friendly measures, such as waste management or energy conservation, in healthcare settings. Students' attitudes and perceptions towards sustainable development would be better assessed through surveys or questionnaires (Richardson et al. 2016). Sustainable Outcomes are better assessed through the evaluation of the environmental impact of nursing practices before and after the implementation of sustainable education.

## 5 Discussion

This concept analysis contributes an original synthesised perspective advancing coherence on sustainability education specifically tailored for application in undergraduate nursing curriculums. Compared to previous interpretations, the developed conceptualisation provides a more comprehensive framing—encompassing attributes aligned with wider sustainability constructs beyond predominantly environmental components, while still foregrounding the interrelations of human and ecological health.

These findings carry salient implications for informing policies and competency models guiding the integration of sustainability content in nursing programmes. The antecedent factors highlighted reinforce calls to strengthen systems thinking, transdisciplinary collaboration, and sustainability‐focused educational leadership as imperatives to drive sustainability advances in nursing education (Osingada and Porta 2020; Lopez‐Medina et al. 2019). The defining attributes provide a comprehensive understanding of the concept of nursing education for sustainable development, highlighting its ecological, environmental, future‐oriented, global, holistic and maintenance‐focused nature (Anåker and Elf 2014; Şimşek and Erkin 2022). The concept attributes and definitional components similarly provide a strategic framework to guide curriculum development, mapping of sustainability learning objectives, and content delivery models.

The model case exemplifies the application of nursing education for sustainable development by integrating the principles of critical pedagogy and the SDGs into nursing curricula, thereby preparing nursing students to address global concerns and promote sustainability in their practice (Fields et al. 2022; Ward et al. 2022; Cook et al. 2019; Leffers et al. 2017). The borderline case depicted in this study highlights the importance of not just introducing sustainable development in nursing education, but also integrating it throughout the curriculum to ensure that nursing students develop a holistic understanding of its principles and their application to various aspects of nursing practice (Şimşek and Erkin 2022; Fields et al. 2022). In a similar vein, our contrary case highlights the need for a shift in nursing education to prioritise sustainable development principles and equip nursing students with the skills and knowledge necessary to address these global concerns in their practice.

Additionally, the multi‐level consequences suggest potential impact at individual competency, healthcare delivery and broader health system levels—directing attention to opportune areas for sustainability education evaluation research. Specifically, longitudinal and interventional designs tracking sustainability competency progression and associated clinical practice impacts will continue adding clarity (Richardson et al. 2016; Walpole et al. 2019). Qualitative case studies detailing curriculum innovation processes also can elucidate best practices for sustainability education design in nursing contexts. Collectively, such research can further validate and refine core conceptual components elucidated through this foundational analysis.

The empirical referents of the concept of nursing education for sustainable development include the integration of sustainability into nursing academic programmes, the description of the academic subject of nursing, and its incorporation into national and international healthcare organisations (Şimşek and Erkin 2022). This concept is also recognised as a key aspect for healthcare professionals to take action towards achieving the targets of the SDGs, requiring active participation in social affairs to promote a culture of sustainability both professionally and personally.

## 6 Key Points

Based on this comprehensive concept analysis, we deduce that nursing education for sustainable development is fundamentally about:

- Training future nurses to understand the deep interconnections between human health, community wellbeing, and environmental sustainability. It moves beyond viewing health solely in individual terms to an eco‐social lens.
- Integrating sustainability principles throughout nursing curriculums and clinical experiences, not siloed in isolated lessons. This enables development of sustainability competencies applicable across diverse practice settings.
- Cultivating key skillsets like systems thinking and change management to graduate sustainability‐minded nurses prepared to spearhead positive impacts in healthcare.
- Framing sustainability as an issue of equity and social justice given disproportionate climate and environmental burdens on marginalized groups. Nurses can address these disparities through sustainable, ethical care.
- Empowering nurses to partner with communities in finding solutions that jointly foster healthy people and healthy environments, embracing nurses' roles as change agents who can bridge environmental and public health advocacy.

## 7 Conclusion

Overall, this analysis makes a significant research contribution through its advancement of conceptual coherence, specificity and applicability to inform sustainability education in nursing programmes. Finally, the concept evaluation presents a springboard to inform nascent evaluation research on learning processes and clinical practice impacts resulting from nursing sustainability education innovations. Collectively, this can galvanise momentum for nursing schools to deliver graduates capable of embedded sustainability application in their vital role nourishing human and environmental health.

## Author Contributions

**Dorothy Serwaa Boakye**, **Atswei Adzo Kwashie**, **Samuel Adjorlolo** and **Kwadwo Ameyaw Korsah:** conceptualised the study, developed the methods, drafted the original manuscript and contributed equally to the editing and review of the manuscript. All authors have approved the published version of this paper.

## Ethics Statement

The authors have nothing to report.

## Consent

The authors have nothing to report.

## Conflicts of Interest

The authors declare no conflicts of interest.

## Data Availability

The data that support the findings of this study are available on request from the corresponding author. The data are not publicly available due to privacy or ethical restrictions.
